# *TIDDIT*, an efficient and comprehensive structural variant caller for massive parallel sequencing data

**DOI:** 10.12688/f1000research.11168.2

**Published:** 2017-06-30

**Authors:** Jesper Eisfeldt, Francesco Vezzi, Pall Olason, Daniel Nilsson, Anna Lindstrand

**Affiliations:** 1 Department of Molecular Medicine and Surgery, Karolinska Institutet, 171 76 Stockholm, Sweden; 2 Center for Molecular Medicine, Karolinska Institutet, 171 76 Stockholm, Sweden; 3 Science for Life Laboratory, Karolinska Institutet Science Park, 171 21 Solna, Sweden; 4 Department of Biochemistry and Biophysics, Stockholm University, 171 21 Stockholm, Sweden; 5 Science for Life Laboratory, Dept of Cell and Molecular Biology, Uppsala University, Husargatan 3, Uppsala, SE-752 37, Sweden; 6 Department of Clinical Genetics, Karolinska University Hospital, 171 76 Stockholm, Sweden

**Keywords:** Variant calling, Whole genome sequencing, Structural variation

## Abstract

Reliable detection of large structural variation ( > 1000 bp) is important in both rare and common genetic disorders. Whole genome sequencing (WGS) is a technology that may be used to identify a large proportion of the genomic structural variants (SVs) in an individual in a single experiment. Even though SV callers have been extensively used in research to detect mutations, the potential usage of SV callers within routine clinical diagnostics is still limited. One well known, but not well-addressed problem is the large number of benign variants and reference errors present in the human genome that further complicates analysis. Even though there is a wide range of SV-callers available, the number of callers that allow detection of the entire spectra of SV at a low computational cost is still relatively limited.

## Introduction

Genomic structural variants (SVs) are defined as large genomic rearrangements and consist of inversion and translocation events, as well as deletions and duplications ^1^. SVs have been shown to be both the direct cause and a contributing factor in many different human genetic disorders, as well as in more common phenotypic traits ^2– 4^.

In genetic diagnostics, current techniques such as FISH ^5^ and microarray studies ^6^ have limited resolution, and the information obtained often needs to be complemented by additional methods for a correct interpretation ^5, 6^. Previous studies have shown that whole genome sequencing can be used to successfully identify and characterize structural variants in a single experiment ^7^.

The advent of systems like the Illumina HiSeqX allows researchers to sequence Whole Human Genomes (WHG) at high ( *i.e.*, 30X) coverage and at a relatively low cost ( *i.e.*, 1000$ per WHG) ^8^. The ability to produce large amounts of data at an unprecedented speed has initiated a flourishing of computational tools that are able to identify ( *i.e.*, call) structural variants and/or chromosomal breakpoints ( *i.e.*, the exact position in the chromosome at which SV takes place). These tools are commonly called *variant callers*, or simply, *callers*. Variant callers generally require an alignment file (in BAM format) as input and try to identify differences between the reference genome and the donor/patient genome. To detect structural variants, callers generally use heuristics based on different signals in the WGS data. These signals include discordant pairs ^9, 10^, read-depth ^11^, and split-reads ^12^. Some callers try to reconstruct the patient sequence by applying either local ^13^ or global ^14^ *de novo* assembly techniques. Depending on the size, variant type, and characteristics of the sequencing data, the most suitable method for detecting a variant will differ ^15^.

Thanks to the ability to produce high quality sequencing data at a relatively low cost, as well as the potential to detect any variant from a single experiment, whole exome sequencing ^16, 17^ and whole genome sequencing ^7, 18^ could be highly useful in clinical diagnostics, especially to study rare disease causing variants. However, to avoid high validation costs, highly precise, yet sensitive callers are needed. To further complicate the situation, an abundance of sequencing platforms and library preparation methods are available ^19^. Sequencing data generated from these different sources have different properties, such as read length and coverage ^20^. As an example, it has been shown that large insert mate pair libraries are well suited to detect SVs ^21^, mainly due to the ability to span repetitive regions and complex regions that act as drivers of structural variation ^22^ and due to the sensitivity derived from a large physical span coverage compared to small insert size sequencing coverage.

Here we present a new variant caller, TIDDIT. The name highlights the ability to detect many different types of SVs; including but not restricted to translocations, inversions, deletions, interspersed duplications, insertions and tandem duplications. TIDDIT utilizes discordant pairs and split reads to detect the genomic location of structural variants, as well as the read depth information for classification and quality assessment of the variants. By integrating these WGS signals, TIDDIT is able to detect both balanced and unbalanced variants. Finally, TIDDIT supports multiple paired-end sequencing library types, characterized by different insert-sizes and pair orientations.

To simplify the search for rare disease causing variants, TIDDIT is distributed with a database functionality dubbed SVDB (Structural Variant DataBase). SVDB is used to create structural variant frequency databases. These databases are queried for rare disease causing variants, as well as variants following certain inheritance patterns. Utilizing the database functionality, the analysis of rare variants may be prioritized, thus speeding up the diagnosis of rare disease causing variants. To our knowledge, no available caller provides such an extensive framework to call and evaluate rare disease causing structural variants.

## Methods

### Implementation

***Detection of structural variants.*** TIDDIT requires a coordinate-sorted BAM file as input. There are two phases; in the first phase, coverage and insert size distribution are computed from the BAM file. These data will be used in the subsequent phase. In the second phase, TIDDIT scans the BAM file for discordant pairs and split reads and uses these signals to detect and classify structural variants. These two signals are pooled together by redefining each signal as two genomic coordinates *S _i_* = ( $p_{i}^{1}$, $p_{i}^{2}$), for a split read the $p_{i}^{1}$ position is given to the position of the primary alignment, and the $p_{i}^{2}$ position is given to the position of the supplementary alignment of that read. On the other hand, for discordant pairs, the $p_{i}^{1}$ position is given to the the read having the smallest genomic coordinate, and the $p_{i}^{2}$ position is given to the position of the read that has the largest genomic coordinate. A read pair is deemed to be discordant if the reads map to different contigs, or if the distance exceeds a threshold distance, *T _d_*. By default, *T _d_* is set to three times the standard deviation plus the average value of the insert size distribution. Every time a SV signal is identified, TIDDIT switches from reading-mode to discovery-mode. As soon as the signal *S _i_* = ( $p_{i}^{1}$, $p_{i}^{2}$) is identified, the set *D* is initialized:

$$D=\{(p_{1}^{1},p_{1}^{2})\}\;(1)$$

At this point TIDDIT searches for other evidence in the neighborhood. Every time a new signal is identified it is added to set *D*. The construction of this set is halted only when no other signal is identified in *W* consecutive positions. The parameter *W* is set to two distinct values, one value when adding split reads to the cluster, and one value when adding discordant pairs. TIDDIT switches through these two settings automatically depending on which signal that is being added. When adding discordant pairs to a possible cluster, *W* is set to 48 * $\sqrt{mean\;insert\;size}$. The square root of the mean insert size was found to be effective for scaling *W* for usage with both small and large insert size libraries. The default value of *W* was found by benchmarking TIDDIT on the sequencing data of more than 350 known SV carriers, sequenced through various libraries. The insert sizes of these libraries were 350 bp, 3 kb, and 20 kb. For split reads, *W* is set to the average read length. The user may change these settings to fine tune the analysis.

In more detail, if ( $p_{1}^{1}$) = *c _j_* ( *i.e.*, $p_{1}^{1}$ is a position *j* on chromosome *c*), then *D* will contain the following signals:

$$D=\{(p_{z}^{1},p_{z}^{2})\}:p_{z}^{1}>c_{j}\wedge$$

$$\;\exists \{(p_{k}^{1},p_{k}^{2}):k\neq z\wedge p_{k}^{1}>c_{j}\wedge p_{z}^{1}<p_{k}^{1}+W\}\;(2)$$

The first condition guarantees that *D* will contain only signals found after position *c _j_*, *i.e.*, the position of the first signal within the set *D*. The existential clause guarantees that the set *D* will not contain any signal that is too far away from the signals within the set *D*. *D* is obtained by reading the ordered BAM file and populating a data structure with all the detected discordant pairs and split reads. If no signal is identified after reading *W* positions from the last position containing a read added to *D*, the discovery phase is closed. TIDDIT also records information about local coverage and read orientation while constructing *D*. Once *D* is computed, TIDDIT partitions it into distinct sets *D*_1_, *D*_2_, ..., *D _k_* such that:

$$\forall i,j\in \{1\ldots \ldots k\}:\left|D_{i}\cap D_{j}\right|=0<$$

$$if\;u_{j}=min\{p_{z}^{2}:p_{z}^{2}\in D^{j}\}$$

$$\text{then}\;D^{i}=\{(p_{z}^{1},p_{z}^{2}):(p_{z}^{1},p_{z}^{2})\in D\wedge (\exists (p_{k}^{1},p_{k}^{2}:k\neq z\wedge$$

$$\;p_{k}^{1}>u_{j}\wedge p_{k}^{2}-W<p_{z}^{2}<p_{k}^{2}+W)\}\;(3)$$

In other words, *D* is divided into non overlapping partitions, *D _k_* requiring that all *p*_2_ positions form a cluster with properties analogous to the *p*_1_ positions. Once *D* is divided into partitions, TIDDIT checks if any partition represents a structural variant or if it is only noise. In the case of it being a structural variant, TIDDIT tries to associate the identified signal with a specific type of variation. A set *D _k_* is discarded ( *i.e.*, the SV is not reported) if the number of pairs forming the set ( *i.e.*, the cardinality of the set) is below a given threshold. This threshold is used to control the sensitivity and specificity of TIDDIT. In general, the number of discordant pairs is dependent on multiple factors, and may vary considerably throughout the genome. Therefore the user may need to fine tune the required number of discordant pairs based on the downstream analysis. All callable structural variants in *D* are reported, and thereafter, *D* is discarded. TIDDIT will then return to read mode, starting with the next available read pair.

***Classification of structural variants.*** TIDDIT identifies candidate variations using discordant pairs and split reads. To determinate the variant type, TIDDIT analyses read orientation, as well as the coverage across the region of the first reads, second reads, and the region in between. TIDDIT characterizes three levels of coverage: low, normal and high. If $\tilde{C}$ is the average coverage computed over the entire genome sequence, then the coverage across a region, *C* is deemed normal if it is satisfying the following condition:

*P* ← *The ploidy of the organism*

$$\left(1-\frac{0.7}{P})\leq \frac{C}{\tilde{C}}\leq (\frac{0.7}{P}+1)\;(4\right)$$

**Figure 1.**
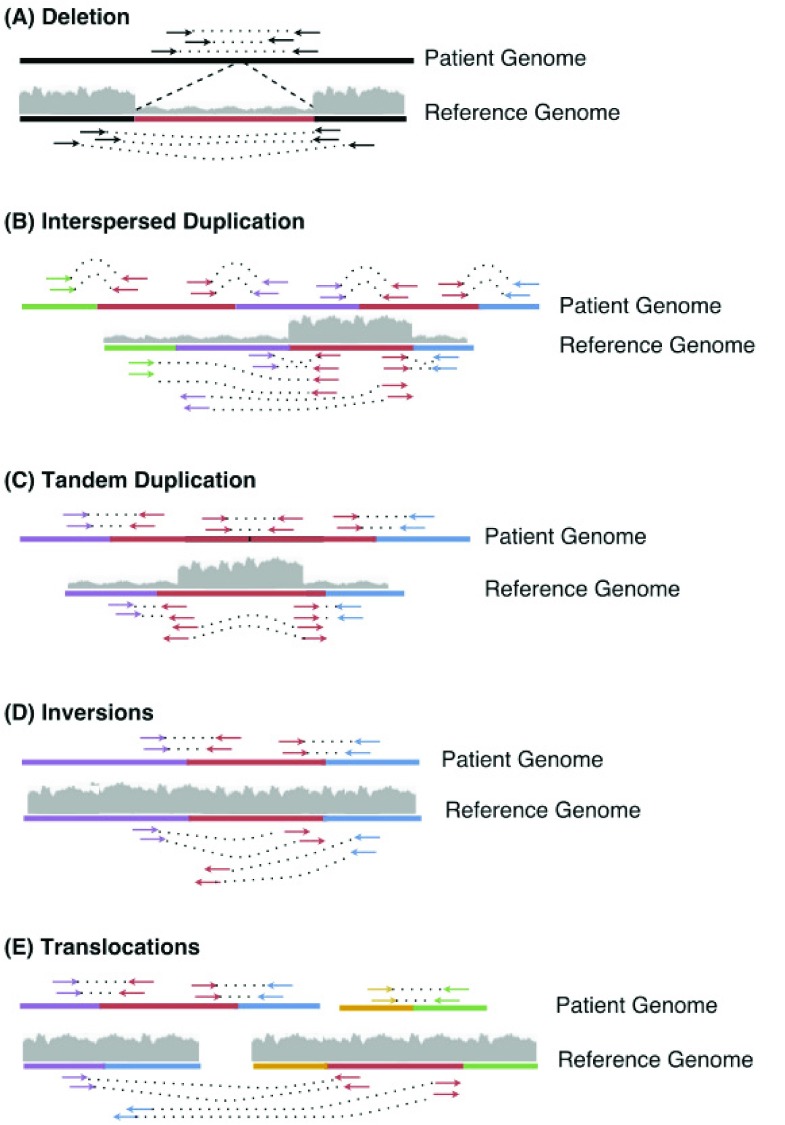
A summary of the SV detected by TIDDIT, and how these SV are classified. TIDDIT classifies the following SV; Deletions ( **A**), Interspersed duplications ( **B**), Tandem duplications ( **C**), Inversions ( **D**), and translocations ( **E**). The arrows indicate the reads and their orientation, where reads mapping along the + strand is indicated by arrows pointing to the right, and reads mapping along the - strand points to the left. In this image, the standard library orientation is forward-reverse. The coverage of a region is indicated by the gray area.

If the coverage across a region is lower than normal, it is classified as low coverage. Likewise, if the coverage is higher than normal coverage, it is classified as a high coverage region. The patterns of the variants detected by TIDDIT are represented in Figure 1.

In a deletion event ( Figure 1A) a region is absent in the patient genome but present in the reference. When aligned to the reference, the read pairs flanking the deleted region will have a larger insert size than what is expected based on the library insert size distribution. Moreover, split reads will be formed in such a way that one part of the read is aligned to one side of the breakpoint, and another part of the read will be aligned to the other side of the deletion. Furthermore, the coverage of the lost region will be lower than expected. Therefore, TIDDIT will classify a variant as a deletion if the region flanked by the discordant pairs/split reads is classified as a low coverage region.

In an interspersed copy number event ( Figure 1B), an extra copy of a region within the genome (the red sequence in Figure 1B) is positioned distant from the original copy. In this case, there will be read pairs and reads bridging the duplicate copy and the distant region. When aligned to the reference, the read pairs will appear to have unexpectedly large insert size, and the reads will appear split across the reference. Thus, TIDDIT will detect these signals. Furthermore, the coverage of the copied region will be higher than expected. By scanning the coverage of the regions where the reads of the discordant pairs are located, TIDDIT will find which region has an extra copy. The interspersed duplication is reported as two events, an intrachromosomal translocation between the duplicated region and the position of the copy, and a duplication across the duplicated region.

In a tandem duplication event ( Figure 1C), the extra copy is positioned adjacent to the original copy. Since the distance between the segments is small, there will be pairs where one read is located at the end of the original copy, and the other read is located at the front of the duplicate copy ( Figure 1C). When aligned to the reference, the insert size of these read pairs will be as large as the tandem copy itself ( Figure 1C). Similarly, there will be reads bridging the two copies. These reads will appear split when mapped to the reference. Furthermore, the orientation of these read pairs and split reads will be inverted. Moreover, the coverage across the entire genomic region will be higher than expected. Thus to classify tandem duplications, TIDDIT will search for sets of discordant pairs/split reads that have inverted read pair orientation as well as high coverage between the read pairs.

In an inversion event ( Figure 1D), the sequence of a genomic region is inverted. When aligning the sequencing data the insert size of the read pairs bridging the breakpoints will appear to be larger than expected. Furthermore, both read pairs will get the same orientation, such as reverse/reverse or forward/forward. TIDDIT employs the read pair orientation in order to identify inversions. Given that the inversion is large enough, TIDDIT will find the pairs bridging the breakpoints of the inversion, and will classify the variant as an inversion if the orientation of the discordant pairs are forward/forward or reverse/reverse orientation, and if the orientation of the primary/secondary alignments are forward/reverse, reverse/forward.

In an interchromosomal translocation event ( Figure 1E), the first read and second read will map to different contigs ( Figure 1E). Reads bridging the translocated segment will appear split between these two contigs. Any read pair mapping to two different contigs is counted as a discordant pair, and any set of signals mapping to different contigs will be classified as an interchromosomal translocation. Intrachromosomal translocation events are similar. They are balanced events, where a genomic region has been translocated to another location within the same chromosome. When aligned to the reference region, these variants will give rise to signals where one read is mapping to the translocated region, and the other read mapping to the region where the translocated region is positioned. This will give rise to pairs having larger insert size than expected. However, since there is no change in copy number, the coverage will be normal across the discordant pairs. The orientation of the reads forming the discordant pairs will depend on whether the translocated region is inserted in its original orientation, or if it is inverted relative to its original orientation. Thus the read pairs may retain the standard library orientation, but the orientation could also be inverted. Therefore, intrachromosomal translocations are classified by scanning for discordant pairs having either forward/reverse or reverse/forward orientation and normal coverage in the region between those reads.

***Filtering of structural variant calls.*** For each called variation, several statistical values are calculated. They serve two purposes: to provide more information and to filter out noise. In the former case, statistics are employed to understand the structure of the variant and to relate it to the rest of the genome. In the latter case, filters are employed to improve the precision of TIDDIT. TIDDIT utilizes four complementary filters: *Expected*_ *links*, *Few*_ *links*, *Unexpected*_ *coverage*, and *Smear*. These heuristics are used to set the FILTER column of the VCF generated by TIDDIT.

The main goal of *Expected*_ *links* is to filter variants caused by random events such as contamination or sequencing errors. It uses a statistical model to compute the expected number of discordant pairs ^23^ using the library insert size, read length, ploidy of the organism and coverage across the region affected by the structural variant. A variant that is defined by less than 40% of the expected number of pairs will fail the *Expected*_ *links* quality test, and is set to *Expected*_ *links* in the FILTER column of the VCF. The statistical model supports variants that are called using discordant pairs, hence for calls based on split reads exclusively, the number of split reads divided by coverage and ploidy is used as an estimate of the expected number of split reads.

*Few*_ *links* aims to filter out calls that are caused by reference errors and misalignment of repetitive regions. As mentioned previously, a variant is defined as a set of positions, $D_{k}=(p_{z}^{1},p_{z}^{2}).$ In order to compute the *Few*_ *links* filter, for each *D _k_*, TIDDIT creates another set called *D _Spurr_*, containing spurious read pairs. Spurious reads pairs are pairs that belong to the interval identified by *D _k_*, but whose mates align to a different chromosome from the one where the pairs form *D _k_*. In other words, TIDDIT checks if a genomic location where a SV can be called is linked to multiple other events. In this case the suspicion is that the called SV is the consequence of a repetitive element. If the fraction of spurious read pairs is too high, the variant within *D _k_* is considered unreliable, and thus its filter flag is set to *Few*_ *links*. The fraction of spurious read pairs is considered too high if the following formula holds true:

*P* ← *The ploidy of the organism*

$$\frac{\left|D_{k}\right|}{\left|D_{spur}\right|+\left|D_{k}\right|}>\frac{0.8}{p}\;(5)$$

*Smear* is a filter designed to remove variants called due to large repetitive regions. In these regions, the split reads and discordant pairs will map in a chaotic manner, hence these regions may appear to be affected by large structural variation. These calls are recognized by searching for variants where the regions of the first read and its mate overlap, or where the regions of the primary and secondary alignment overlap. If this is the case, the variants will fail the *Smear* test. The filter flag of variants that fail this test is set to *Smear*.

Lastly, *Unexpected*_ *coverage* also aims to filter out calls caused by reference errors and misalignments, but unlike the *Few*_ *links*, it employs coverage information. The *Unexpected*_ *coverage* filter uses the coverage across the region of the *p*_1_ signals as well as the region of *p*_2_ to determine the quality of a variant call. If the coverage of any of these regions is 10 or more times higher than the average library coverage, the variant will fail the *Unexpected*_ *coverage* test, and its FILTER column is set to *Unexpected*_ *coverage*.

Any variant that passes these filters is set to PASS, those that fail are set according to the filter that rejected the variant. By removing all variants that did not pass this quality control, the precision of TIDDIT improves considerably.

### Structural variant database

Using the structural variant frequency database (SVDB) software, the user may compare variants found in different samples and annotate the VCF files with the frequency of each variant. The frequency database is built with multiple VCF files containing structural variant information. The VCF files may be generated using any caller that reports structural variants according to the VCF standard. By removing high frequency variants from the VCF file, rare variants may be detected. The database could also perform trio analysis and filter out variants following a certain frequency pattern within a family.

The database is an SQLite database. It contains one entry per input variant. These entries describe the genomic position, variant type, and sample id of the variant. Moreover, each variant is given a unique index number. The database software extracts the variant type from the *ALT* field. Once constructed, these SQLite databases may be used either directly to provide frequency annotation, or they may be exported to a VCF file.

***Export.*** The variants of the SQLite structural variant database may be exported to a VCF file. The VCF file is a standard structural variant VCF file, generated by clustering similar variants within the SQLite database, and reporting each cluster as a single VCF entry. The INFO field of each VCF entry contains four custom tags. The FRQ tag describes the frequency of the variant, the OCC tag describes the total number of samples carrying the variant, the NSAMPLES tag describes the total number of samples within the database, and lastly, the variant tag describes the position and id of each clustered variant.

The clustering of the variants is performed using one out of two methods, either an overlap based clustering method or DBSCAN ^24^.

***Annotation.*** The main purpose of the structural variant frequency database is to query it and use it for frequency annotation. The frequency database is queried using a VCF file. All variants within the VCF file will be annotated with the frequency of that variant within the frequency database. The database used for querying may either be an SQLite database, an exported VCF file, or a multi-sample VCF file such as the thousand genome structural variant VCF.

If an SQLite database is chosen, two separate algorithms are available, overlap or DBSCAN. If DBSCAN is chosen, all variants of the SQLITE database and the query VCF are clustered using DBSCAN. Thereafter, the frequency of each query variant is set to the number of separate samples represented in the cluster of that query variant.

When querying the database(either a SQLite database, or vcf), a query may be similar to multiple database entries. If this is the case, the frequency of the variant will be based on the total number of individuals carrying those variants.

***DBSCAN clustering.*** DBSCAN is one of the two algorithms available through the SVDB package. DBSCAN requires two parameters, epsilon and minPTS ^24^. Epsilon is the maximum breakpoint distance between the cluster and a variant that is to be addded, and minPTS is the minimum number of variants needed to form a cluster. At default epsilon is set to 500 bases, and minPTS is set to 2 samples. However, these parameters may be changed by the user.

The DBSCAN clustering is performed by dividing each chromosome and variant type into separate sub-databases; thereafter, a 2-dimensional coordinate system is defined for each sub-database. For intrachromosomal variants, the x coordinate of this plane corresponds to the start position of the variant, and the y coordinate within the plane corresponds to the end position of the variant.

Interchromosomal translocations involve two chromosomes, so when clustering these variants, the contig id is sorted according to lexicographic order. Out of the two contigs involved in the rearrangement, the contig ordered first is set to the x axis, and the contig last in order is set to be the y axis. Thereafter, each variant is added to the plane as described for intrachromosomal variants. This procedure is repeated for any possible chromosome pair, and each variant type on each chromosome pair.

Once each plane is defined, the variants within each separate plane are clustered using DBSCAN.

***Overlap based clustering.*** The most critical part when building and querying the database is to determine if two SVs represent the same event or not. When using the overlap based clustering algorithm, two interchromosomal variants are considered equal if the distance between their breakpoints does not exceed a certain distance threshold. This distance is set to 5 kilobases (kB) as default. However, the user may change it to suit any kind of data.

Additionally, for intrachromosomal variants, the overlap *O* is computed and used to determine if the variants are similar enough. For a given chromosome, to compute *O* each variant *var* is regarded as an ordered set of genomics coordinates:

*var* = { *i*, *i* + 1,..., *j* — 1, *j*}                                                            (6)

The overlap parameter *O* is defined as the cardinality of the intersection of two variants, divided by the cardinality of the union of the same overlapping variants:

$$\;O=\frac{\left|var_{1}\cap var_{2}\right|}{\left|var_{1}\cup var_{2}\right|}\;(7)$$

Where *var*_1_ and *var*_2_ are two overlapping variants ( *O* equals to 0 if the variants are not overlapping). The default threshold value of the overlap parameter is 0.6.

Variants from different individuals overlap in complex patterns. When these variants are clustered and exported into a vcf file, the complex patterns needs to be resolved. Resolving the complex overlaps correctly is of great importance: firstly because the user may choose to use the exported database for frequency annotation, and secondly because the similarity between the query variant and cluster will depend on how the cluster is represented. The database functionalities of SVDB solves this problem by finding a balance between compression ( *i.e.*, the number of clusters), and resolution ( *i.e.*, how close these clusters resemble the variants of the exported database). The final goal is to represent the population within the database in a way which is as useful as possible for downstream analysis (for example to characterize variants which are common within a population).

**Figure 2.**
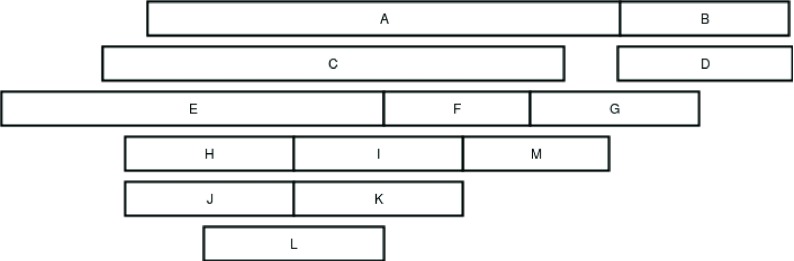
An example of a complex region, this regions contains 13 variants, some of which are very similar (such as *B* and *D*), while others are entirely different ( *A* and *B*), when exporting the database, these variants needs to be reported in a way that is meaningful to the user.

In the example shown in Figure 2 There are 13 variants that are to be exported using the overlap based clustering method. Initially, the variants are clustered using a greedy approach; these clusters are formed by first picking a random variant, and thereafter expanding the cluster iteratively by adding variants which are similar to any other variant in the cluster. In the example shown in Figure 2, a cluster is initiated by picking variant A. variant C is similar to A, hence it is added to the cluster. Next the cluster is expanded by searching for variants similar to C. In this example, variant E will be added to the cluster, since it satisfies the similarity criteria with C. E is not similar to any variant not belonging to the cluster, hence the expansion of the cluster will be halted.

The same procedure is repeated for all other variants, for this example, all other variants will be added into one cluster, variant H and J is similar to L, which is similar to I and K, which in turn are similar to F; F is similar to M, and M is similar to G, which is similar to B and D.

Next, SVDB aims to dissect these clusters into sub-clusters for a more coherent representation of the variants. The clusters are divided into sub-clusters by computing a connectivity matrix. This connectivity matrix is a *n* ∗ *n* matrix, where *n* is the number of variants within the cluster. For variants satisfying the similarity criteria (breakpoint distance, and overlap of intrachromosomal variants), the matrix entry is set to 1, otherwise, the entry is set to 0. In practice, the matrix is implemented as a sparse matrix. An example of the connectivity matrix of the cluster containing variants B, D, F, G, H, I, J, K, L, M of Figure 2 is given in Table 1.

**Table 1.** A connectivity matrix computed using the overlap based clustering method to the complex cluster of variants shown in Figure 2. This cluster contain variant B,D,F,G,H,I,J,K,L, and M.

A SVDB connectivity matrix										
	*B*	*D*	*F*	*G*	*H*	*I*	*J*	*K*	*L*	*M*
***B***	1	1	0	1	0	0	0	0	0	0
***D***	1	1	0	1	0	0	0	0	0	0
***F***	0	0	1	0	0	1	0	0	0	1
***G***	1	1	0	1	0	0	0	0	0	1
***H***	0	0	0	0	1	0	1	0	1	0
***I***	0	0	1	0	0	1	0	1	1	0
***J***	0	0	0	0	1	0	1	0	1	0
***K***	0	0	0	0	0	1	0	1	1	0
***L***	0	0	0	0	1	1	1	1	1	0
***M***	0	0	1	1	0	0	0	0	0	1

Thereafter the sub-clusters are defined. The sub-clusters are generated by treating the variants as nodes in a graph. For each node ( *i.e.*, variant) within the graph, the number of edges represent the number of variants satisfying the similarity criteria.

A sub-cluster is defined by selecting the node in the graph which has the highest degree, all nodes directly connected to this node will belong to that sub-cluster. Thereafter the sub-cluster is reported to the vcf file (in the manner described under the export section), the sub-cluster is represented by the variant (node) having the highest connectivity, and the frequency of that variant will be based on the variants connected to it.

Once the sub-cluster is reported, all variants (nodes) belonging to that sub-cluster is marked as unavailable; among the remaining nodes, the software will pick the node with the highest degree, and the process is repeated. Once no node is available, all variants of the cluster are represented in the vcf, and the software will continue on to the next cluster; dissecting it into sub-clusters which satisfy the user set parameters.

**Figure 3.**
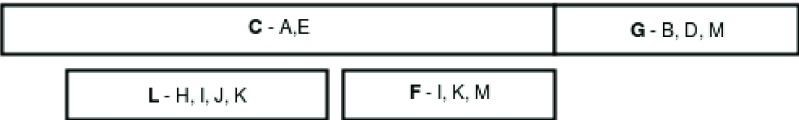
The results of clustering the variants shown in Figure 2. The clusters are represented by the bold variant shown in front of the "-", while the variants shown after the "-" belong to the cluster. As shown, a variant may belong to multiple clusters.

By clustering the variants of Figure 2 we obtain the clustering represented in Figure 3. We must point to the fact that the results of clustering will depend on which parameters are used. In this case, an overlap of 0.5 was used. Figure 2 shows variants represented in multiple clusters.

When a query is found to overlap a cluster, the overlap is reported together with the number of original variants overlapping the query. Moreover, if a query matches multiple overlapping clusters, each single variant will be counted only once (as a consequence of how the clusters are constructed).

### Operation

***Detection of structural variants.*** TIDDIT requires a coordinate sorted BAM file as input, and may be run in two separate modes; variant calling or coverage computation. The coverage computation mode is used to compute the coverage across the entire genome, and returns a BED file as output. The variant calling mode is run to analyse SV across the entire genome. All the detected SVs are returned in a single VCF file.

***System requirements.*** TIDDIT has been tested on a large number of datasets; In general, TIDDIT will perform variant calling in less than 2 hours ( Table 4) using a single CPU core, and 2 gigabytes of RAM memory on 30X human whole genome datasets (such as NA12878). The time consumption and memory usage is mainly dependent on the coverage and quality of the input data. TIDDIT has been tested on Linux as well as Apple macOS. TIDDIT is easy to install and requires only standard c++ libraries. TIDDIT is installed using cmake ( https://cmake.org/) and make ( https://www.gnu.org/software/make/).

***Downstream analysis.*** TIDDIT generates a VCF file containing structural variant calls across the entire genome. Initially, these calls may be filtered based on the quality filters described in the implementation section, as well as the SVDB software, using either internal samples or an external dataset such as thousand genomes structural variants ^25^ ( ftp://ftp.1000genomes.ebi.ac.uk/vol1/ftp/phase3/integrated_sv_map/). The TIDDIT output VCF is compatible with any tool which supports VCF, hence there’s a large number of tools available for further analysis of the variant calls. Typically, the VCF file is annotated using software such as VEP ^26^ or snpEFF ^27^. Tools such as VCFtools ^28^ and BEDtools ^29^ may be used to remove low quality calls, or filter calls within specific genomic regions.

## Results

The performance of TIDDIT was evaluated using simulated, as well as public datasets containing a large number of validated variants of different size and type. The performance of TIDDIT was compared to current top of the line callers, including Lumpy ^30^, Delly ^10^, CNVnator ^11^, Manta ^9^, and Fermikit ^14^. These are well known callers that have been tested throughout numerous projects.

Three separate datasets were used to evaluate TIDDIT. First, a simulated dataset generated using Simseq ^31^ and SVsim ( https://github.com/gregoryfaust/svsim) was used. Next, two public large scale sequencing datasets were used, namely the NA12878 sample, and the HG002 sample ^32^. Truth sets of validated variants were found for each of the public datasets ^32, 33^. A detailed description of the benchmarking of TIDDIT is given in Supplementary File 1.

### Performance of TIDDIT on simulated variants

To test the performance of the callers, the tools were run on the simulated dataset. The dataset consists of four separate samples, one per variant type. The coverage of these simulated samples was set to 25X, and the read length and insert size was set to 100 and 350 bp, respectively. For each variant type (deletions, duplications, inversions, translocations), 6000 variants were simulated. Hence, the simulated dataset contains 24000 known variants of different type. The results are presented in Table 2. For each caller the sensitivity and precision was computed. The sensitivity differs between different callers and variant types. Table 2 shows that TIDDIT is consistently the caller with the highest sensitivity. However, the margin of victory is relatively small compared to the second-most sensitive callers. And even though TIDDIT provides high precision calling (around 0.99 for most SV-types), the precision of some of the other callers is slightly higher. Hence, there is a trade-off between sensitivity and precision, and it is not evident which caller is performs the best on this dataset. Looking more closely at the translocations in Table 2 we can see that TIDDIT has a good trade-off between sensitivity and precision, revealing the highest sensitivity out of all callers, and precision that was only 0.02 lower than Delly and Manta.

**Table 2.** Sensitivity and precision of the structural variant callers on a simulated dataset consisting of 6000 variants of each variant type. The variants were simulated using SVsim and Simseq.

SV detection on simulated data				
Caller	Sensitivity	Precision	Sensitivity	Precision
	**Deletions**		**Duplications**	
TIDDIT	0.96	0.99	0.96	0.99
CNVnator	0.9	0.92	0.86	0.91
Delly	0.94	1	0.95	1
Fermikit	0.41	1	0.33	1
Lumpy	0.95	0.97	0.95	1
Manta	0.95	1	0.95	1
	**Inversions**		**Translocations**	
TIDDIT	0.97	0.99	0.92	0.93
Delly	0.94	1	0.87	0.95
Fermikit	0.35	1	0.26	0.99
Lumpy	0.5	1	0.87	0.9
Manta	0.95	1	0.88	0.95

### Performance of TIDDIT with NA12878 and HG002

Lastly, to complement the benchmarking on the simulated dataset, and to test TIDDIT on a more diverse set of sequencing data, the same callers were run on the NA12878 and HG002 samples ^32^. The results are presented in Table 3. We computed both sensitivity and precision, in three size intervals. Events 0–100 bp in size are usually not considered structural variation, but they were kept as a size interval anyway since some callers also detect these variants. Briefly, the NA12878 sample was sequenced using a 30X paired 2X150 bp library, while the HG002 sample was sequenced using a 6KB insert size mate-pair library.

**Table 3.** The sensitivity (S) and precision (P) of six structural variant callers with the public NA12878 and HG002 samples. These public datasets contain validated deletions of various sizes.

Detection of validated deletions in public data sets						
Size(bp)	0–100		100–1000		≥ 1000	
	S	P	S	P	S	P
**NA12878**						
TIDDIT	0.55	0.7	0.78	0.71	0.98	0.55
CNVnator	0	0	0.1	0.3	0.55	0.17
Delly	0.62	0.11	0.84	0.63	0.96	0.41
FermiKit	0.03	0.2	0.64	0.68	0.56	0.7
Lumpy	0.59	0.21	0.81	0.5	0.96	0.5
Manta	0.91	0.01	0.9	0.43	0.95	0.45
**HG002**						
TIDDIT	0.09	0.95	0.49	0.78	0.53	0.33
CNVnator	0	0	0.02	0.45	0.52	0.1
Delly	0.1	0.46	0.29	0.7	0.45	0.35
FermiKit	0	0	0	0.67	0	0.5
Lumpy	0.04	0.58	0.3	0.8	0.33	0.2

With the NA12878 sample, the performance differed widely between callers and variant sizes ( Table 3). With variants larger than 1000 bp, TIDDIT had the highest detection rate and second best precision. FermiKit was the most precise tool, but it must be noted that such result was achieved at the expense of sensitivity, since Fermikit had almost half the sensitivity of TIDDIT. In other words, FermiKit was not able to call many true variants, but the variants that were called were more likely to be correct. On the other hand, TIDDIT was able to call almost all the validated large ( *i.e*. *≥* 1000 bp) variants, but many calls done by TIDDIT did not overlap with the validated ones. However, since the truthset only contains high quality deletion calls, these non-overlapping calls are not necessarily incorrect. With medium ( *i.e.*, 100 *−* 1000 bp) and small ( *i.e.*, *≤* 100 bp) size variants the performance of the callers differed greatly. For these variants, Manta had the highest sensitivity, while TIDDIT had the highest precision. In general, when working with real data the differences in performance between the tools is less evident if compared to simulated data. This is likely a consequence of the fact that most of the tools have been extensively tested and, to some extent, tuned on these public datasets. Moving on to the HG002 sample, it was found that most variant callers performed worse than with the NA12878 sample. No variant caller produced any significant number of true positive calls in the range of 0–100 bp. TIDDIT had the highest sensitivity on variants larger than 100 bases, and was one of the most precise callers ( Table 3). Manta was excluded from this benchmark since it does not support mate-pair libraries.

### CPU hour consumption of the variant callers

The computational performance of the six tools was also determined. Despite not being the most important parameter to consider, CPU time rising in importance, especially if one needs to run analysis in a Compute Infrastructure as a Service ( *e.g.*, Google Cloud or Amazon). The CPU hour consumption of the callers was measured while analyzing each sample. The results of the measurements are presented in Table 4. During the analysis, each caller except Fermikit was run on a single core of an Intel Xeon E5-2660 CPU, and Fermikit was run using 16 Intel Xeon E5-2660 CPU cores.

**Table 4.** CPU hour consumption of the structural variant callers. Each caller except Fermikit was run on a single core of a Intel Xeon E5-2660 CPU. Fermikit was run on 16 CPU cores. The CPU hour consumption of the Simseq data is reported as the median time consumption across the four Simseq samples.

CPU hour consumption on SV calling			
Caller	NA12878	HG002	Simseq
TIDDIT	2	1	1
CNVnator	2	1	1
Delly	30	15	7
FermiKit	640	120	15
Lumpy	45	2	7
Manta	3	NA	1

It was found that CNVnator and TIDDIT are the most efficient callers, while FermiKit is by far the most expensive caller to run.

***Evaluation of the database functionality***. The performance of the database functionality was evaluated by building databases of different sizes. These databases were built by randomly sampling individuals from a set of 209 individuals, sequenced through the thousand genomes project ^25^. These individuals are listed in Supplementary File 1.

**Figure 4.**
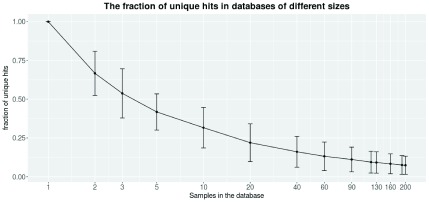
As the number of samples within the database increase, the number of unique variants per sample decrease.

Figure 4 shows how the fraction of unique hits within the database gets lower as the size of the database increases, therefore improving the ability to find unique variants for new patients. A unique hit is defined as a variant that has only been found in the query itself. Already, a relatively small database filtered out a significant amount of variants. On average, a sample queried against a database consisting of only 10 samples contain about 25% unique variants. Still, a larger database filters out more variants. Each query sample was found to contain 7.5% ( *i.e.*, *∼*250) unique structural variants when filtered against a database containing 200 samples. Since each caller reports a relatively large fraction of false positives ( Table 3), the frequency database is necessary to reduce the number of variants. Moreover the frequency database can be use to filter out recurring technical errors connected to the library preparation, sequencing chemistry and alignment of the sequencing data.

**Figure 5.**
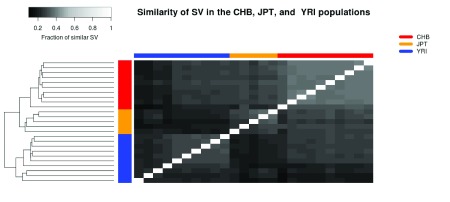
A comparison of SV calls between twenty five individuals, belonging to the JPT, CHB and YRI populations. These individuals were sequenced through the thousand genomes project. The heatmap is coloured based on the similarity between individuals, and shows that the populations can be distinguished based on their SVs.

The structural variant databases may also be used to benchmark different tools and settings, as well as to compare the SVs within a family or population. As an example, the database functionality was used to study differences between populations sequenced through the thousand genomes project ^25^. Three populations were selected; Han Chinese from Bejing (CHB), Japanese from Tokyo (JPT), and Yoruba from Ibadan (YRI). Twenty five samples was analysed in total, 10,5, and 10 samples from the CHB, JPT, and YRI populations, respectively. All these samples were analysed using TIDDIT. The similarity of the samples was determined by creating one database per sample, and querying each sample against each database. The fraction of similar SVs was determined by computing the number of common/similar SVs divided by the total number of SV in that sample. A more detailed description of the analysis and the selected samples is given in Supplementary File 1. It was found that the three populations are distinct based on their SVs. Furthermore, the CHB and JPT populations are relatively similar compared to the YRI population ( Figure 5). The CHB population appears to be more homogeneous than the other two populations, with the exception of one individual which appears to be similar to the JPT individuals. On the other hand, the YRI population appears to be most diverse population, and is divided into two clear subpopulations.

## Discussion

### Variant calling

Six structural variant callers were benchmarked on simulated data generated by simseq ( Table 2), and two public datasets, including NA12878 and HG002 ( Table 3). Compared to the other callers, TIDDIT performs well on SV larger than 1000 bp ( Table 2, Table 3). TIDDIT is able to identify large structural variants in many experimental setups: low or high coverage, short or long fragment size ( *i.e.*, Paired End or Mate Pair) ( Table 2– Table 3). Furthermore, TIDDIT has a good balance between sensitivity and precision. Despite being one of the most sensitive tools, it is also one of the most precise tools. These two characteristics (high sensitivity and high precision) in conjunction with a low computational demand makes TIDDIT one of the most capable tools available today for the identification of large SVs greater than 1 kbp from WGS data. TIDDIT does not perform well on small variants ( Table 3), however TIDDIT performs really well on large variants, especially balanced variants ( Table 2). Since TIDDIT is efficient, produces high quality variant calls, and performs well in multiple settings, TIDDIT could be a great addition to WGS pipelines.

### Benchmarking

Even though the presented benchmarking is extensive, it is not fully complete. The public datasets lack large enough truth sets for balanced variants and duplications. Since the callers perform differently on different variants ( Table 2), it would be of value to benchmark the callers against a more varied set of variants. Moreover, the variants of these truth sets are generally small. For instance, the median size of the deletions of NA12878 is about 250 bases, which is smaller than the traditional size of structural variation ^3^. Unlike real SV which is commonly located in repeat sequences, the simulated SV were positioned randomly throughout the genome. This could partly explain why the sensitivity of all callers is much higher on the simulated datasets compared to the public datasets ( Table 2, Table 3). Ideally, the simulated variants should follow the true characteristics of SV. Since such characteristics to a certain degree are unknown, but also to simplify the simulation, it was decided to generate a randomly uniform dataset.

### Database functionality

The Human genome contains a large amount of repetitive regions, and each individual carries a large number of structural variants ^34, 35^. Due to these reasons, the number of detected structural variants per samples is generally high. Thus, finding a rare disease-causing variant among such a large number of common variants is difficult and time consuming. TIDDIT is distributed together with structural variant database software. This software package uses structural variant VCF files to construct variant frequency databases. The annotation provided by these databases is then used to filter common variants as well as reference errors.

By filtering out high frequency variants from the VCF file, rare disease causing variants can easily be detected ( Figure 4). Moreover, the database could be constructed to follow variants that all samples have in common, such as an inherited disease variant within a family, or known disease causing variants, as well as to search for variants following a certain inheritance pattern, or to compare the SVs of different populations ( Figure 5). The structural variant databases could also be used to benchmark different software tools, settings, and library preparation methods. The database functionality can be used for results obtained from any tool that generates a valid VCF file as format.

### Database clustering algorithms

Clustering of SV is not a novelty, a wide range of clustering algorithms has been applied to cluster various SV types generated from various data ^25, 36^, commonly these software tools are developed and used inhouse ^37^. Compared to many of these techniques, SVDB allows clustering of all types of SV, generated via a wide range of methods (as long as the SV is presented in vcf format). Moreover, SVDB supports a wide range of operations, including building, and querying of databases. SVDB comes with two separate but related clustering algorithms, namely overlap based clustering and DBSCAN. The two clustering methods will produce the same clusters for some variants, and different clusters in other cases. One notable difference is that the overlap based method will require some degree of overlap between the variants of a cluster, while DBSCAN only requires that the breakpoints of the variants are closer than the user set epsilon distance.

Additionally, when opting for DBSCAN the user can set the minPTS parameter to require a minimum number of variants supporting a cluster, as well as to decide the compactness of a cluster. Conversely, this is not possible when opting for the overlap clustering method.

In short, the two methods of SVDB are similar, Which one to pick depends largely on how the user defines the similarity of SV, and which questions the user needs to answer when using the software.

## Conclusions

TIDDIT is an efficient and comprehensive structural variant caller, supporting a wide range of popular sequencing libraries. Not only does TIDDIT have the functionality of a structural variant caller, it also has a set of functions that helps the user perform further analysis of the bam file. These functions include depth of sequencing coverage analysis and structural variant database functionality. By utilizing these functions, TIDDIT could either perform advanced analysis on it’s own or be used to perform a wide range of tasks within a variant analysis pipeline. TIDDIT has already been employed in many studies and demonstrated its potential not only with the commonly used Nextera mate pair libraries from Illumina ^7, 18, 38^ but also with the TrueSeq Nano and PCR-free Paired End libraries ^39^.

## Software availability

Latest source code: https://github.com/J35P312/TIDDIT

Archived source code as at the time of publication: https://zenodo.org/account/settings/github/repository/J35P312/TIDDIT

License: GNU General Public License version 3.0 (GPLv3)

## Supplementary material

Supplementary File 1: Supplementary methods. A description of the benchmarking of the SV callers.

Supplementary File 2: SV simulation pipeline. The pipeline used to generate the simulated SV datasets.
