# Supplementary material for: TIDDIT, an efficient and comprehensive structural variant caller for massive parallel sequencing data
Source: F1000Res. 2017 Jun 30;6:664. Originally published 2017 May 10. [Version 2] doi: 10.12688/f1000research.11168.2 (PMC5521161; doi:10.12688/f1000research.11168.2)
Supplement: Supplementary file 1 [file f1000research-6-13002-s0000.tgz › 88aee70a-7a62-48aa-8499-e4e00688348c.pdf]

TIDDIT, an efficient and comprehensive structural variant caller for massive parallel sequencing data: Supplementary material

Jesper Eisfeldt [1,2,,*], **Francesco Vezzi** [3,4,*] **,Pall Olason** [5] **,Daniel Nilsson** [1,2,3,6,*,], **and Anna Lindstrand** [1,2,6, †]

[*]Equal Contribution
[†]to whom correspondence should be addressed

Supplementary methods

Benchmarking

The performance of TIDDIT was evaluated using simulated as well as public datasets containing a large number of validated variants of different size and type. The performance of TIDDIT was compared to current top of the line callers, including Lumpy [1], Delly [2], Cnvnator [3], manta [4], and Fermikit [5]. These are well known callers that have been tested throughout numerous projects.

0.1 SV simulation pipeline

The following section explains the pipeline used to generate the simulated data. The reads of the simulated data was generated using Simseq [6], and the variants were simulated using SVsim [7]. The simulation was performed by generating one BAM file per variant type, the simulated variants were deletions, duplications, inversions and translocations. Hence, four separate BAM files were created.

Each simulated BAM file contains 6000 variants. These 6000 variants were simulated by generating two haplotypes with 3000 variants each. The size of the variants were uniformly spread in the range of 1kb-151kb. The sequencing depth of each haplotype was set to 12.5X. These two haplotypes were then merged, producing a diploid sample with coverage of 25X. The read length and insert size was set to 100 bp and 350 bp, respectively. The following procedure was executed for each simulated haplotype:

1. SVsim variant simulation
   (a) input: hg19 reference fasta file, SVSIM config file, seeded with a random number
   (b) output: permuted hg19 reference fasta, tab file containing the position of simulated SVs
2. SimSeq read simulator
   (a) input: permuted hg19 reference fasta file, library settings, Simseq standard error profile
   (b) output: Simseq SAM file

The SAM files of the two haplotypes were then merged using SAMtools merge, and converted into a BAM file using SAMtools view [8]. Next, to produce the four BAM files the following procedure was applied:

1. SAMtools sort and SAMtools bam2fq
   (a) input: merged SimSeq BAM file
   (b) output: interleaved readname sorted fastq
2. BWA-MEM alignment
   (a) input: interleaved readname sorted fastq, hg19 reference fasta file
   (b) output: BAM file aligned using BWA

Lastly, each BAM file was indexed using SAMtools. The script used to run this pipeline is available as Supplementary File 2 (simulate_data.sh), along with the four SVsim config files(haplotype_1_del. txt,haplotype_1_dup.txt, haplotype_1_inv. txt,haplotype_1_tra.txt). Visit the commented lines within each script for more information on how to run the pipeline.

0.2 Availability of public samples and validated variants

Three public datasets were used to complement the simulated dataset. These datasets consists of the NA12878 and HG002 samples, made public via the GIAB consortium [9]. As well as a subset of the thousand genomes project dataset [10].

The downsampled NA12878 paired end BAM was downloaded via this FTP:
`ftp://ftp-trace.ncbi.nlm.nih.gov/giab/ftp/data/NA12878/NIST_NA12878_HG001_HiSeq_300x/`
The truth set of NA12878 is available through the SVclassify manuscript:
`ftp://ftp-trace.ncbi.nlm.nih.gov/giab/ftp/technical/svclassify_Manuscript/Supplementary_Information/`

The HG002 6kB mate pair BAM file was downloaded via the following FTP:
`ftp://ftp-trace.ncbi.nlm.nih.gov/giab/ftp/data/AshkenazimTrio/HG002_NA24385_son/NIST_Stanford_Illumina_6kb_matepair/bams/`
The truth set of HG002 is available through the GIAB FTP:
`ftp://ftp-trace.ncbi.nlm.nih.gov/giab/ftp/data/AshkenazimTrio/analysis/NIST_DraftIntegratedDeletionsgt19bp_v0.1.8/`
The 2 tech filtered noTRs files were used as a truth set.
More information and more data is available upon browsing the FTP.
The HG002 6kb mate pair BAM was converted to fastq using SAMtools bam2fq, and then aligned to hg19 using BWA-MEM.[11].

The thousand genomes bam files were retrieved via the following FTP:
`ftp://ftp.1000genomes.ebi.ac.uk/vol1/ftp/phase3/`
More information on these files are found on the FTP as well as the thousand genomes article [10].

0.3 Callers and settings

TIDDIT was compared to five of the current top of the line callers, including Lumpy [1], Delly [2], Cnvnator [3], manta [4], and Fermikit [5]. All these callers, as well as TIDDIT, were run using their default settings as presented in the manual of each caller. The following versions of the callers were used:

1. CNVnator v0.3.2
2. Delly v0.7.2
3. Fermikit r178
4. Lumpy 0.2.12
5. Manta 1.0.3
6. TIDDIT 2.0

The callers were run using the following commands:

0.3.1 CNVnator

The following command was issued to run CNVnator. $1 is the input BAM file, $3 is the prefix of the output file, and $2 is the path to a directory containing the reference files.

```
cnvnator -root $3.root -tree $1
cnvnator -root $3.root -his 100 -d $2
cnvnator -root $3.root -stat 100 » $3.cnvnator.log
cnvnator -root $3.root -partition 100
cnvnator -root $3.root -call 100 > $3.out
cnvnator2VCF.pl $3.out > $3.cnvnator.vcf
```

0.3.2 Delly

The following command was issued to run delly. $1 is the input BAM file, $3 is the prefix of the output file, and $2 is the reference fasta file.

```
delly -t TRA -o $3.tra.vcf -g $2 $1
delly -t DEL -o $3.del.vcf -g $2 $1
delly -t DUP -o $3.dup.vcf -g $2 $1
delly -t INV -o $3.inv.vcf -g $2 $1
vcf-concat $3.tra.vcf $3.del.vcf $3.dup.vcf $3.inv.vcf > $3.dellySV.vcf
```

0.3.3 fermikit

The following command was issued to run fermikit. $1 is the input BAM file, $3 is the prefix of the output file, and $2 is the reference fasta file. The -l parameter is the read length which was set according to the read length of the sequencing library.

```
samtools bam2fq $1 > $3.fastq
fermi2.pl unitig -s3g -t16 -l100 -p $3 $3.fastq > $3.mak
make -f $3.mak
run-calling -t16 $2 $3.mag.gz | sh
```

0.3.4 Lumpy

The following command was issued to run Lumpy via lumpy express. $1 is the input BAM file and $2 is the prefix of the output vcf.

```
samtools view -b -F 1294 $1 > $2_normal.D.unsorted.bam
samtools sort $2_normal.D.unsorted.bam $2_normal.D
samtools view -h $1 | $extractSplitReads -i stdin | samtools view -Sb - > $2_normal.S.unsorted.bam
samtools sort $2_normal.S.unsorted.bam $2_normal.S
alias awk=gawk
lumpyexpress -B $1 -S $2_normal.S.bam -D $2_normal.D.bam -o $2.lumpy.vcf
```

0.3.5 Manta

The following command was issued to run Manta. $1 is the input BAM file, $3 is the name of the output directory, and $2 is the reference fasta file.

```
configManta.py --normalBam $1 --reference $2 --runDir $3
python $3/runWorkflow.py -m local
```

0.3.6 TIDDIT

The following command was issued to run TIDDIT. $1 is the input BAM file and $2 is the prefix of the output vcf.

```
./TIDDIT --sv -b $1 -o $2
```

0.4 Similarity of variants in the truth sets and called variants

Two different measurements were used to compute the similarity of variants in the truth sets and called variants.
The similarity of translocations was assessed by computing the distance between the breakpoints of the truth set and the variants called by the tools. For the simulated translocations, the breakpoint distance limit was set to 1 kilobases (kB). For all other variants, the similarity between calls and truth sets was computed using the formula presented in the overlap computation section. For all datasets, variants whose overlap ratio exceed 0.6 were considered the same.
Each call was only allowed to generate one hit, i.e if one call satisfies the overlap of multiple variants of the truth set, it will still only count as one hit. Prior to computing the precision and sensitivity of the samples, all low quality calls from all variant callers were removed. These were defined as calls whose vcf quality entry was not set to "Pass" or ".".

0.5 Evaluation of the structural variation database software SVDB

SVDB was presented in two separate scenarios; first as a frequency filter, and secondly as a tool used to compare the SVs of different individuals/settings.
Prior to the SVDB analysis, a dataset consisting of 209 thousand genomes project samples were analysed using TIDDIT. TIDDIT was run using the default settings. Thereafter, all non-PASS variants were removed, and the break-end variants were filtered so that each variant call was represented by one vcf-entry only.
To demonstrate the performance of SVDB on databases of different sizes, the resulting vcf files were analysed using the following command:

```
svdb --hist --sample_hist --n 10 --k 1 5 10 20 40 60 90 120 130 160 190 200 –folder unique/
```

Where unique is the folder containing all the vcf files. Using this command, SVDB will generate databases of each size k (1,5,10,20,40,60,90,120,130,160,190,200), picking vcf files randomly from the input folder. SVDB will generate 10 databases of each size k. Each database is queried using every sample which it consists of. Thereafter, SVDB reports the distribution of the number of unique variants per sample for each k.

Lastly SVDB was used to compare the SVs of the CHB, JPT, and YRI populations. To do so, 25 vcf files were selected from the previously described 209 files. Prior to the SVDB analysis, all variants involving the decoy contig was removed. Thereafter, the samples were compared using the following command:

```
svdb --hist --similarity_matrix --folder input_vcf > populations.txt
```

The resulting output was clustered and visualized using the heatmap.2 function of the R package gplots `https://CRAN.R-project.org/package=gplots`.

0.6 List of thousand genomes samples

The low coverage WGS data of the following samples was used to evaluate SVDB:
NA18486 NA18488 NA18489 NA18498 NA18499 NA18501 NA18502 NA18504 NA18505 NA18507 NA18508 NA18510 NA18511 NA18516 NA18517 NA18519 NA18520 NA18522 NA18523 NA18525 NA18526 NA18528 NA18530 NA18531 NA18532 NA18533 NA18534 NA18535 NA18536 NA18537 NA18538 NA18539 NA18541 NA18542 NA18543 NA18544 NA18545 NA18546 NA18547 NA18548 NA18549 NA18550 NA18552 NA18553 NA18555 NA18557 NA18558 NA18559 NA18560 NA18561 NA18562 NA18563 NA18564 NA18565 NA18566 NA18567 NA18570 NA18571 NA18572 NA18573 NA18574 NA18577 NA18579 NA18582 NA18591 NA18592 NA18593 NA18595 NA18596 NA18597 NA18599 NA18602 NA18603 NA18605 NA18606 NA18608 NA18609 NA18610 NA18611 NA18612 NA18613 NA18614 NA18615 NA18616 NA18617 NA18618 NA18619 NA18620 NA18621 NA18622 NA18623 NA18624 NA18625 NA18626 NA18627 NA18628 NA18629 NA18630 NA18631 NA18632 NA18633 NA18634 NA18635 NA18636 NA18637 NA18638 NA18639 NA18640 NA18641 NA18642 NA18643 NA18644 NA18645 NA18646 NA18647 NA18648 NA18740 NA18745 NA18747 NA18748 NA18749 NA18757 NA18853 NA18856 NA18858 NA18861 NA18864 NA18865 NA18867 NA18868 NA18870 NA18871 NA18873 NA18874 NA18876 NA18877 NA18878 NA18879 NA18881 NA18907 NA18908 NA18909 NA18910 NA18912 NA18915 NA18916 NA18917 NA18923 NA18924 NA18933 NA18934 NA18939 NA18940 NA18941 NA18942 NA18943 NA18944 NA18945 NA18946 NA18947 NA18948 NA18949 NA18950 NA18951 NA18952 NA18953 NA18954 NA18956 NA18957

NA18959 NA18960 NA18961 NA18962 NA18963 NA18964 NA18965 NA18966 NA18967 NA18968 NA18969 NA18970 NA18971 NA18972 NA18973 NA18974 NA18975 NA18976 NA18977 NA18978 NA18979 NA18980 NA18981 NA18982 NA18983 NA18984 NA18985 NA18986 NA18987 NA18988 NA18989 NA18990 NA18991 NA18992 NA18993 NA18994 NA18995 NA18997 NA18998 NA18999

These samples were selected since they were readily available on the computational cluster used during this study.

References

[1] Ryan M Layer, Colby Chiang, et al. Lumpy: a probabilistic framework for structural variant discovery. *Genome biology*, 15(6):1–19, 2014.

[2] Tobias Rausch, Thomas Zichner, et al. Delly: structural variant discovery by integrated paired-end and split-read analysis. *Bioinformatics*, 28 (18):i333–i339, 2012.

[3] Alexej Abyzov, Alexander E Urban, et al. Cnvnator: an approach to discover, genotype, and characterize typical and atypical cnvs from family and population genome sequencing. *Genome research*, 21(6):974–984, 2011.

[4] Xiaoyu Chen, Ole Schulz-Trieglaff, et al. Manta: rapid detection of structural variants and indels for germline and cancer sequencing applications. *Bioinformatics*, page btv710, 2015.

[5] Heng Li. Fermikit: assembly-based variant calling for illumina resequencing data. *Bioinformatics*, page btv440, 2015.

[6] Sam Benidt and Dan Nettleton. Simseq: a nonparametric approach to simulation of rna-sequence datasets. *Bioinformatics*, 31(13):2131–2140, 2015.

[7] Gregory Faust. Gregoryfaust/svsim, Jul 2015. URL `https://github.com/gregoryfaust/svsim`.

[8] Heng Li, Bob Handsaker, et al. The sequence alignment/map format and samtools. *Bioinformatics*, 25(16):2078–2079, 2009.

[9] Justin M Zook, David Catoe, et al. Extensive sequencing of seven human genomes to characterize benchmark reference materials. *Scientific data*, 3, 2016.

[10] 1000 Genomes Project Consortium et al. A global reference for human genetic variation. *Nature*, 526(7571):68–74, 2015.

[11] Heng Li. Aligning sequence reads, clone sequences and assembly contigs with bwa-mem. *arXiv preprint arXiv:1303.3997*, 2013.
