# Supplementary material for: TIDDIT, an efficient and comprehensive structural variant caller for massive parallel sequencing data
Source: F1000Res. 2017 Jun 30;6:664. Originally published 2017 May 10. [Version 2] doi: 10.12688/f1000research.11168.2 (PMC5521161; doi:10.12688/f1000research.11168.2)
Supplement: Peer review history

# Peer review: Referee response for version 2

**DOI:** 10.5256/f1000research.13002.r23932

**Published:** 2017-07-20

**Authors:** Bud Mishra

**Affiliations:** 1 Department of Computer Science, Courant Institute of Mathematical Sciences, New Yok, NY, USA

I have read this submission. I believe that I have an appropriate level of expertise to confirm that it is of an acceptable scientific standard.

# Peer review: Referee response for version 2

**DOI:** 10.5256/f1000research.13002.r23933

**Published:** 2017-07-12

**Authors:** Giuseppe Narzisi, Wayne Clarke

**Affiliations:** 1 New York Genome Center, New York, NY, USA

We thank the authors for addressing our comments.  

 We have no further major comment at this time, just a couple of minor typos listed below:

- Page 8: Once the sub-cluster is reported, all variants (nodes) belonging to that sub-cluster [is -> are] marked as unavailable.
- Page 9: and it is not evident which caller [DELETE: is] performs the best on this dataset.
- Page 10: Twenty five samples [was -> were] analysed in total

We have read this submission. We believe that we have an appropriate level of expertise to confirm that it is of an acceptable scientific standard.

# Peer review: Referee response for version 1

**DOI:** 10.5256/f1000research.12049.r23324

**Published:** 2017-06-26

**Authors:** Bud Mishra

**Affiliations:** 1 Department of Computer Science, Courant Institute of Mathematical Sciences, New Yok, NY, USA

The paper has already two reviewers and they have addressed many of the issues that I would have raised. My main concern however is that the interpretation of the results depend highly on the accuracy of the reference used and that the human reference is still incomplete (non-haplotypic), error-ridden, and evolving. It is unclear how the tool will adapt to the future when large number of accurate haplotypic whole genome references will become available. What makes it even worse is that the software is built on a group of intuitively justifiable heuristics with many hyper-parameters hard-coded. However, all the other tools it competes against also suffer from these problems and the genomics community has been strangely oblivious of these foundational problems.

I have read this submission. I believe that I have an appropriate level of expertise to confirm that it is of an acceptable scientific standard, however I have significant reservations, as outlined above.

# Peer review: Referee response for version 1

**DOI:** 10.5256/f1000research.12049.r22619

**Published:** 2017-06-01

**Authors:** Giuseppe Narzisi, Wayne Clarke

**Affiliations:** 1 New York Genome Center, New York, NY, USA

This paper describes the implementation of a new structural variant caller, called TIDDIT, that uses multiple forms of evidence to call structural variants (deletions, duplications, inversions, and translocations) in whole genome sequencing experiments. In addition to structural variant calling, TIDDIT includes database functionality that helps reduce errors and makes it adaptable to diverse applications (e.g. rare and *de novo* variant detection).

 Structural variant calling is an area of research that is in strong need for more reliable and sensitive bioinformatics methods. TIDDIT has the potential to be a valuable open source tool and, overall, we think that the method warrants publication but some further refinement is necessary. We hope that the authors will address the following major and minor comments:

 Major Comments:

  

1. The authors list the current problems in the field as computational costs, non-standard output formats, and limited support for different sequencing platforms/library types. We don’t believe they have adequately described the tools they compare against with respect to these limitations. For example, both CNVnator and Manta have reasonable computational costs (2 and 3 core hours respectively), while Manta outputs VCF and does not list any platform/library type as a known limitation of their tool. A better description of how their work solves these limitations compared to other tools is required. Alternatively, they could shift focus towards the novelty of the database functionality.
2. Generally, there is sufficient information provided for interpretation. However, there are some cases where further explanations are required. First, Manta is compared against for both the simulated data and NA12878 but is subsequently dropped from analysis of HG002 with no explanation. The authors should provide an explanation for this either in the text or as a footnote to the figures. Second, there is no description of the clustering method selected for the evaluation of database functionality (overlap based and DBSCAN clustering). The two clustering methods are described only from a technical stand point without providing details on how selection would affect the output or what use cases might be appropriate for each method.
3. While the results generally support the conclusions made, the language used tends to overstate the differences between the tested methods: “TIDDIT is consistently the caller with the highest sensitivity”, “The high sensitivity of TIDDIT is coupled with extremely high precision”, “Despite being one of the most sensitive tools, it is also one of the most precise tools”. For example, while it is true that TIDDIT is the caller with highest sensitivity in the simulated datasets (table 1), the margin of victory is often quite low (only 0.01 in deletions and duplications – with lower precision than Manta, the next most sensitive caller). The authors should adjust the language used in the paper to provide a more truthful and honest description of the real performance of the tool as reported in the tables. Significant better performance is indeed achieved for simulated translocations but the improvements on the other classes of variants seem to be limited.
4. We found the description for resolving the chain-like pattern of overlaps in the “overlap based clustering” to be quite confusing and hard to follow as currently described in the paper. The authors should state more clearly the problem. Is this a specific issue that has not been properly addressed by the community so far? Also, it is usually helpful to explain some of the complexity in interval analysis by including figures that elucidate the details of the process.

 

 Minor Comments:

1. The methods used to generate the simulated data is described well enough, however, in this case making the simulated data sets available would also have been practical and would facilitate reproducing the results independently. The code is easily installed and provides sufficient documentation via the help options to get started with using the tool.
2. Simulated structural variants seem to be created at random locations, however real variants tend to happen in a non-random fashion along the genome, in particular around repetitive sequences. It is important to emphasize in the paper the limitations of the simulated data, which may also explain partially why sensitivity on real data is significantly worse than in the simulated experiment. 
3. The caption for Figure 2 seems particularly long for such a simple figure and many of the details given in the figure caption would be better placed in the main text.
4. Equations in the paper should be numbered.
5. On the equation on page 3, the variable *W *is used to indicate the number of consecutive (base pair) positions used to halt the construction of a set. This value seems to play a significant role in partitioning the genome to identify structural variants. However, there is no information on what value is used and whether it is a user parameter.
6. On page 6: “These calls are recognized by searching for variants [were -> where] the regions of the first read and its mate overlap, or where the regions of the primary and secondary alignment overlap.”
7. The authors report TIDDIT’s system requirements to be only 2 hours using a single CPU and 2 GB of RAM. However, these numbers are uninformative without reporting also the amount of data (e.g., sequence coverage, number of reads, etc.) that was used to test the tool.

We have read this submission. We believe that we have an appropriate level of expertise to confirm that it is of an acceptable scientific standard, however we have significant reservations, as outlined above.

# Response

**Published:** 2017-06-22

**Authors:** Jesper Eisfeldt

**Affiliations:** Karolinska Institute, Sweden

Hello there!

 Thanks for taking your time to reviewing, and sorry for taking such a long time to respond! We agree with your comments, and we have incorporated them in the manuscript:

 Major Comments:

 1. We now focus more on the database functionality, but we also mention the number of callers that combine low computational cost and the ability to detect all types of SV is limited.

 2. Manta does not support variant calling on data having read orientation reverse-forward, hence it was excluded from the HG002 dataset which is sequenced using a mate-pair library. We have now added a short description of the two samples, and also a statement why manta was dropped from the analysis of HG002.

 We wanted to use the mate-pair data of HG002 sample mainly to show that TIDDIT performs well on both standard paired-end data and mate-pair data.

 3. Thanks, we have adjusted the language to make it more neutral.

 4. We have rewritten the overlap based clustering section completely, now it contains an example of such complex cluster of variants; as well as a more practical explanation of the algorithm.

 Minor Comments:

 1. Due to the large size of the files we decided not to upload the simulated data. Potentially, we could generate the data and make it available on request during a limited time. We are happy to hear that the code is easily installed!

 2. That’s true, the simulated variants were randomly positioned throughout the genome. We have added some comments to the benchmark subsection of the discussion section were we comment on this. The main reasons we simulated the variants that way was to reduce the risk of any selection bias, as well as to simplify the simulation.

 We agree that the most truthful simulation would include more realistically positioned variants, and perhaps even a large number of known disease causing SV.

 3. We summarized the caption

 4. Thanks, we added numbering to the equations!

 5. We added some details on how W is set; In short, we found it through benchmarking on about 350 WGS samples of patients carrying known SV of clinical relevance, which were sequenced using a variety of libraries. However, since we cannot make the patient data publicly available we cannot present our internal benchmarking in the paper.

 7 We got the statistics from running TIDDIT on the NA12878 sample, we have now added some more detail to that statement.

 Thanks again for reviewing!

 Regards

 Jesper on behalf of the authors
